# Oblique impact responses of Hybrid III and a new headform with more biofidelic coefficient of friction and moments of inertia

**DOI:** 10.3389/fbioe.2022.860435

**Published:** 2022-09-08

**Authors:** Xiancheng Yu, Peter Halldin, Mazdak Ghajari

**Affiliations:** ^1^ HEAD Lab, Dyson School of Design Engineering, Imperial College London, South Kensington, United Kingdom; ^2^ Division of Neuronic Engineering, Department of Biomedical Engineering and Health Systems, KTH Royal Institute of Technology, Huddinge, Sweden; ^3^ MIPS AB, Täby, Sweden

**Keywords:** headform, oblique impact, helmet, brain injury, head injury, rotational acceleration

## Abstract

New oblique impact methods for evaluating head injury mitigation effects of helmets are emerging, which mandate measuring both translational and rotational kinematics of the headform. These methods need headforms with biofidelic mass, moments of inertia (MoIs), and coefficient of friction (CoF). To fulfill this need, working group 11 of the European standardization head protection committee (CEN/TC158) has been working on the development of a new headform with realistic MoIs and CoF, based on recent biomechanics research on the human head. In this study, we used a version of this headform (Cellbond) to test a motorcycle helmet under the oblique impact at 8 m/s at five different locations. We also used the Hybrid III headform, which is commonly used in the helmet oblique impact. We tested whether there is a difference between the predictions of the headforms in terms of injury metrics based on head kinematics, including peak translational and rotational acceleration, peak rotational velocity, and BrIC (brain injury criterion). We also used the Imperial College finite element model of the human head to predict the strain and strain rate across the brain and tested whether there is a difference between the headforms in terms of the predicted strain and strain rate. We found that the Cellbond headform produced similar or higher peak translational accelerations depending on the impact location (−3.2% in the front-side impact to 24.3% in the rear impact). The Cellbond headform, however, produced significantly lower peak rotational acceleration (−41.8% in a rear impact to −62.7% in a side impact), peak rotational velocity (−29.5% in a side impact to −47.6% in a rear impact), and BrIC (−29% in a rear-side impact to −45.3% in a rear impact). The 90th percentile values of the maximum brain strain and strain rate were also significantly lower using this headform. Our results suggest that MoIs and CoF have significant effects on headform rotational kinematics, and consequently brain deformation, during the helmeted oblique impact. Future helmet standards and rating methods should use headforms with realistic MoIs and CoF (e.g., the Cellbond headform) to ensure more accurate representation of the head in laboratory impact tests.

## 1 Introduction

Pedal cyclists and motorcyclists are among the most vulnerable road users. In 2019, 846 pedal cyclists and 5,014 motorcyclists died, and nearly 49,000 pedal cyclists and 84,000 motorcyclists were injured in road traffic collisions in the United States (NHTSA, 2021a; NHTSA, 2021b). In Great Britain in 2019, the casualty rate per billion passenger miles was 4,891 for pedal cyclists and 5,051 for motorcyclists, over 25 times higher than car occupants (Murphy, 2020). In addition, the fatality rate per billion passenger miles in the United Kingdom was 29 for pedal cyclists and 105 for motorcyclists, which were 18 and 65 times higher than car occupants, respectively. A key injury sustained by cyclists and motorcyclists in road traffic collisions is head injury, which has life-threatening and life-changing consequences. Their head injuries can include skull fractures, rupture of superficial vessels, focal damage to the brain tissue, and damage to deep structures of the brain including axons and blood vessels (Chinn et al., 2001; Baker et al., 2022). Helmets are designed to protect cyclists and motorcyclists against head injuries (Ghajari et al., 2011a; Abayazid et al., 2021). Various helmet standards are in place across different countries and regions for assessing the protective performance of helmets (Fernandes and De Sousa, 2013).

Headforms are used in laboratory test methods to evaluate the performance of helmets. The fidelity of physical properties of headforms is crucial to ensure that laboratory tests adequately predict mechanical measures of injury that are expected in real-world collisions under the same impact conditions. Current helmet standards use translational acceleration of the headform to assess the performance of helmets (McIntosh and Grzebieta, 2013; Becker et al., 2015). However, motivated by extensive biomechanics evidence and the development of new helmet technologies (Halldin et al., 2003; Kurt et al., 2017; Bliven et al., 2019; Khosroshahi et al., 2019; Siegkas et al., 2019), new test methods are emerging that require measuring both translational and rotational motions of the headform to evaluate injury criteria, such as peak rotational acceleration and velocity. These kinematic measures are further used to define the loading of finite element models of the human head, which have been widely used to study brain injuries (Ganpule et al., 2017; Ghajari et al., 2017; Fahlstedt et al., 2021; Yu et al., 2022a). The introduction of these new test methods has raised new questions: how does the biofidelity of headforms influence the measured translational and rotational motions? How does it affect predictions of human head FE models, which have not been reported in previous experimental studies (Connor et al., 2018; Trotta et al., 2020; Juste-Lorente et al., 2021)?

The key properties that affect translational and rotational motion of the headform are mass, moments of inertia (MoIs), and coefficient of friction (CoF). However, the EN960 and Hybrid III (HIII) headforms, which are widely used in helmet standards, rating methods, and helmet research (FIM,; Mills, 2010; Stigson et al., 2017; Deck et al., 2021; Yu et al., 2022a), have limitations in relation to one or more of these properties. The EN960 headform was shown to have a larger mass than the human head with the same circumference (Connor et al., 2020). In contrast, the HIII headform was shown to have a more realistic mass (Connor et al., 2018). The MoIs of the EN960 headform were shown to be significantly higher than the human head’s MoIs, calculated from CT data using the same head circumference (Connor et al., 2020). Such large differences in MoIs lead to the large difference in the head response during the oblique impact, suggesting that the EN960 headform is not suitable for oblique impact tests (Connor et al., 2018). The HIII headform was originally developed for vehicle crash tests (Backaitis and Mertz, 1993), with the MoI about the Y axis (left–right) similar to the human head. However, the suitability of this headform for helmet assessment has been criticized due to its unrealistic MoIs about the X (posterior–anterior) and Z (inferior–superior) axes (Yoganandan et al., 2009; Connor et al., 2018; Connor et al., 2020).

The CoF between the headform and helmet liner also plays a key role in determining the head kinematics during the impact. The CoFs between the helmet liner and the EN960 and HIII headforms were measured as 0.16 and 0.75, respectively (Trotta et al., 2018a). The HIII headform has a much higher CoF than the human scalp (Trotta et al., 2018a). A previous study compared the headform response by using the bare EN960 and silicon-covered EN960 headforms and exposing them to the same oblique impact (Ebrahimi et al., 2015). The results showed that the headform–liner CoF plays an important role in headform’s rotational acceleration. These conclusions were further confirmed by a recent study, investigating the headform’s CoF under the oblique impact with varying tangential velocities (Juste-Lorente et al., 2021). Recently, Trotta et al. (2018b) attached a porcine scalp on both EN960 and HIII headforms and compared their kinematics with the bare headforms. The results showed that the porcine scalp reduced both translational and rotational accelerations through energy absorption and sliding. Another recent study showed that the animal scalp also affects the performance of helmet technologies designed for head rotation mitigation (Zouzias et al., 2021).

Despite these attempts on investigating the effects of MoIs or CoF on head rotational kinematics, a key limitation of these studies is that none of them used headforms that have both realistic MoIs and CoF as in the human head. For instance, Connor et al. (2018) 3D-printed a headform with realistic MoIs, based on the geometry of the EN960-M headform, and compared its response to EN960-M, EN960-J, and HIII headforms under the oblique impact, but they did not measure the CoF of the headforms. They found that the 3D-printed headform produced much higher rotational acceleration and velocity than the EN960-M headform under the oblique impact, but it is not clear whether this difference is due to their different MoIs or CoFs, or both. Similarly, other studies have used headforms with realistic CoF but not MoIs (Trotta et al., 2018b; Juste-Lorente et al., 2021; Zouzias et al., 2021). The rotational kinematics of a headform that has both biofidelic MoIs and CoF remains unknown. In addition, it is unclear whether such a biofidelic headform would produce head kinematics that is significantly different from other widely used headforms, particularly the HIII headform.

To address the need for a headform that has both realistic MoIs and CoF, working group 11 (WG11) of the European standardization head protection committee (CEN/TC158) has developed specifications for a new headform based on recent biomechanics research on the mass, center of gravity, moments of inertia, and coefficient of friction of the scalp/helmet interface and the shape of the head. A version of the headform, with both realistic MoIs and CoF, has been prototyped by Cellbond Ltd. This study aims to introduce an early version of this headform, called the Cellbond headform hereafter, and compare its measurements in the helmeted oblique impact with those from the HIII headform, which is widely used for testing helmets under the oblique impact in research studies and rating methods (Stigson et al., 2017; Bliven et al., 2019; Deck et al., 2021; Yu et al., 2022a). It should be noted that this early version is being amended, and hence its final version may have slight differences from the version used here. We test whether there is a difference between the predictions of the Cellbond and HIII headforms in terms of injury metrics based on head kinematics. We also use a finite element model of the human head to predict the strain and strain rate across the brain and test whether there is a difference between the headforms in terms of the predicted strain and strain rate. The results of this study can improve our understanding of the differences between these headforms and support the rationale for including a headform with realistic MoIs and CoF (e.g., the Cellbond headform) in helmet test standards and rating methods. In addition, the test results presented here may be used as a baseline for future studies using other headforms.

## 2 Methods

### 2.1 The moments of inertia and coefficient of friction of the hybrid III and Cellbond headforms and the human head

**FIGURE 1:**
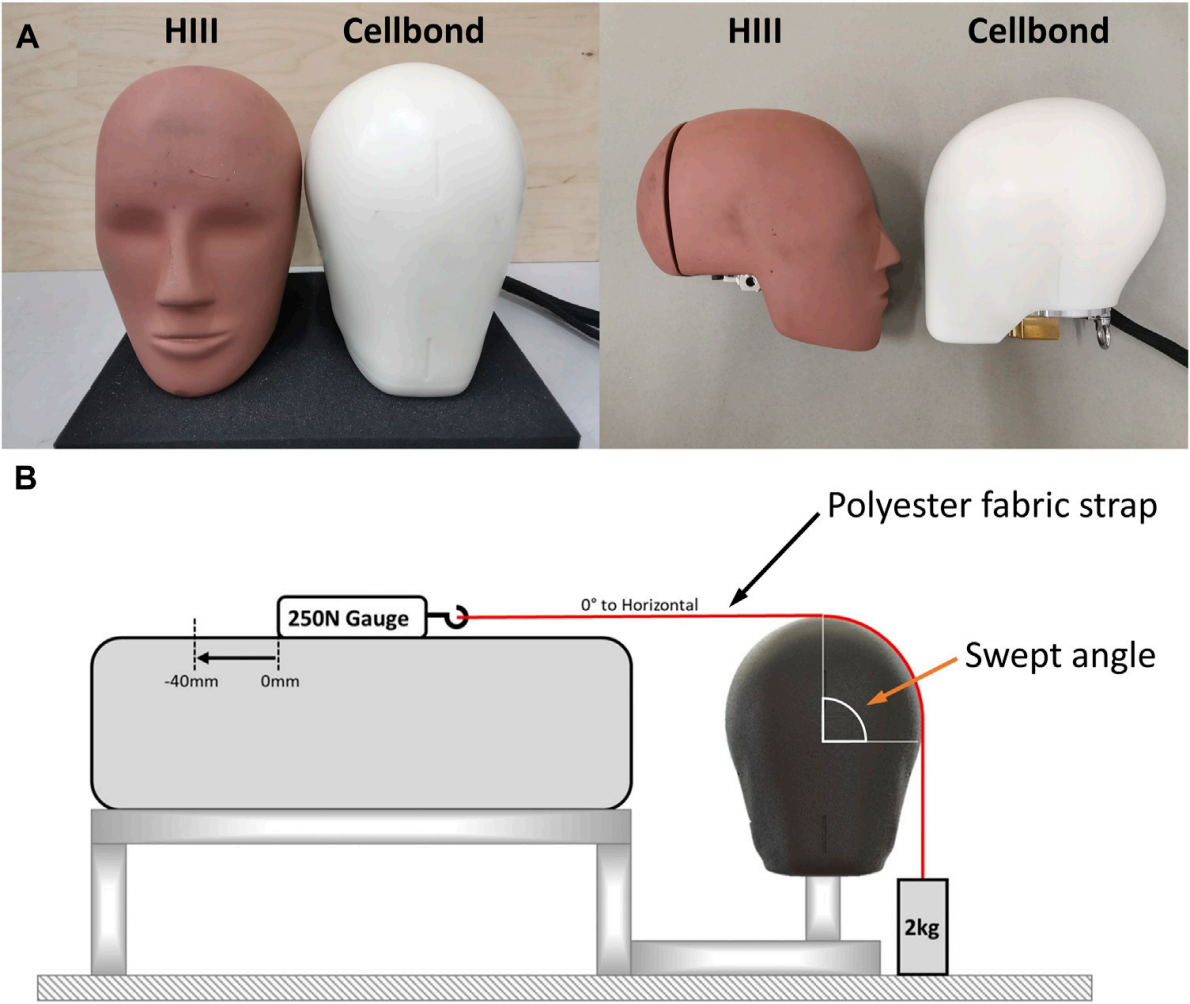
**(A)** Hybrid III headform (left) and the Cellbond headform (right). **(B)** Friction tests for measuring the CoF of headforms.

We used two headforms to conduct the helmet tests (Figure 1A). We used the 50th percentile HIII headform, which had a metal casing covered with a rubber skin. This headform has been widely used for testing helmets, particularly where rotational kinematics of the headform is measured (Aare and Halldin, 2003; Henry et al., 2017; Bliven et al., 2019; Abayazid et al., 2021; Fahlstedt et al., 2021). We also used a new headform manufactured by Cellbond, a division of Encocam Limited, under the instruction of WG11 (Figure 1A). The Cellbond headform is made of Nylon through the injection molding process. A few aluminum plates are fitted inside and at the bottom of the headform for balancing the mass and MoIs and for mounting sensors. The MoIs of the two headforms are presented in Table 1 as the “measured” data.

**TABLE 1:** Physical properties of the headforms and human head.

Physical property	Hybrid III headform		Cellbond headform		CoF of PMHS heads (Trotta et al., 2018a)
	Measured	Calculated	Measured	Calculated	–
Mass (kg)	4.54	4.54	4.41	4.41	–
I_xx_ (kg*cm^2^)	159	230	196	219	–
I_yy_ (kg*cm^2^)	240	243	237	232	–
I_zz_ (kg*cm^2^)	220	158	155	151	–
Scalp–liner CoF	0.75	–	0.18	–	0.2–0.26 with hair; 0.27–0.32 without hair
Skull–scalp CoF	–	–	–	–	0.06

A recent study has determined the relationship between the head mass and MoIs using both cadaveric data and living human computed tomography (CT) scans (Connor et al., 2020). It has shown that CT data have good agreement with the cadaver data, but there is less variation in the CT data. Therefore, we used the mass–MoI relationship based on the CT data (56 adults and 42 males) to calculate the MoIs for the HIII and Cellbond headforms, presented in Table 1 as the “calculated” data. The MoIs of the Cellbond headform are close to the human head data with a 10.5% difference in I_xx_, 2.2% difference in I_yy_, and 2.6% difference in I_zz_. For the HIII headform, the differences are 30.9% (I_xx_), 1.2% (I_yy_), and 39.2% (I_zz_).

We conducted friction tests to measure the CoF of the two headforms (Figure 1B). A polyester strap was fixed to a mass of 2 kg, and the strap was pulled over (up to 40 mm) the surface of the headform at a constant rate of 150 mm/min, with a swept angle of 90°. The force was measured using a 250-N force gauge. The pulling force was averaged between 15 mm and 25 mm pulling distance. The CoF was determined from the capstan equation (Gao et al., 2015):

$$T_{pull}=T_{mass}\cdot e^{\left(f\cdot \theta \right)},$$

where $T_{pull}$ is the pulling force, $T_{mass}$ is the weight of the mass (2 kg), f is the CoF, and θ is the swept angle. The CoFs of the HIII and Cellbond headforms were measured as 0.75 and 0.18, respectively (Table 1).

A recent study on the sliding behavior of the scalp using postmortem human heads has shown that the scalp sliding mainly includes an initial skull–scalp sliding followed by scalp–liner sliding (Trotta et al., 2018a). They have shown that skull–scalp CoF is very low (0.06 ± 0.048), and the stroke of scalp–skull sliding can be larger than 10 mm. They have also found that the dynamic scalp-liner CoF is between 0.2 and 0.32, depending on the sliding direction and presence of hair. The headforms used here, similar to other available headforms, can only mimic the scalp–liner sliding. Compared with the HIII headform, which has a very high CoF, the CoF of the Cellbond headform (0.18) is closer to the average of the CoF values for the skull–scalp and scalp–liner sliding (0.13–0.19).

### 2.2 Oblique impact tests

The oblique impact tests were conducted at the Human Experience, Analysis, and Design (HEAD) lab at Imperial College London. We generally followed the newly introduced oblique impact test method in the ECE22.06 motorcycle helmet test standard, including the helmet fitting, impact velocity, and oblique anvil. (Committee, 2020). The only difference is that ECE22.06 uses the EN960 headform, but here we used the HIII and Cellbond headforms. We did not include the EN960 headform in the tests because doing so would have required designing a new accelerometer mount due to the narrow cavity in this headform compared with the HIII and Cellbond headforms, and that the response of this headform has been studied previously under the oblique impact (Connor et al., 2018).

**FIGURE 2:**
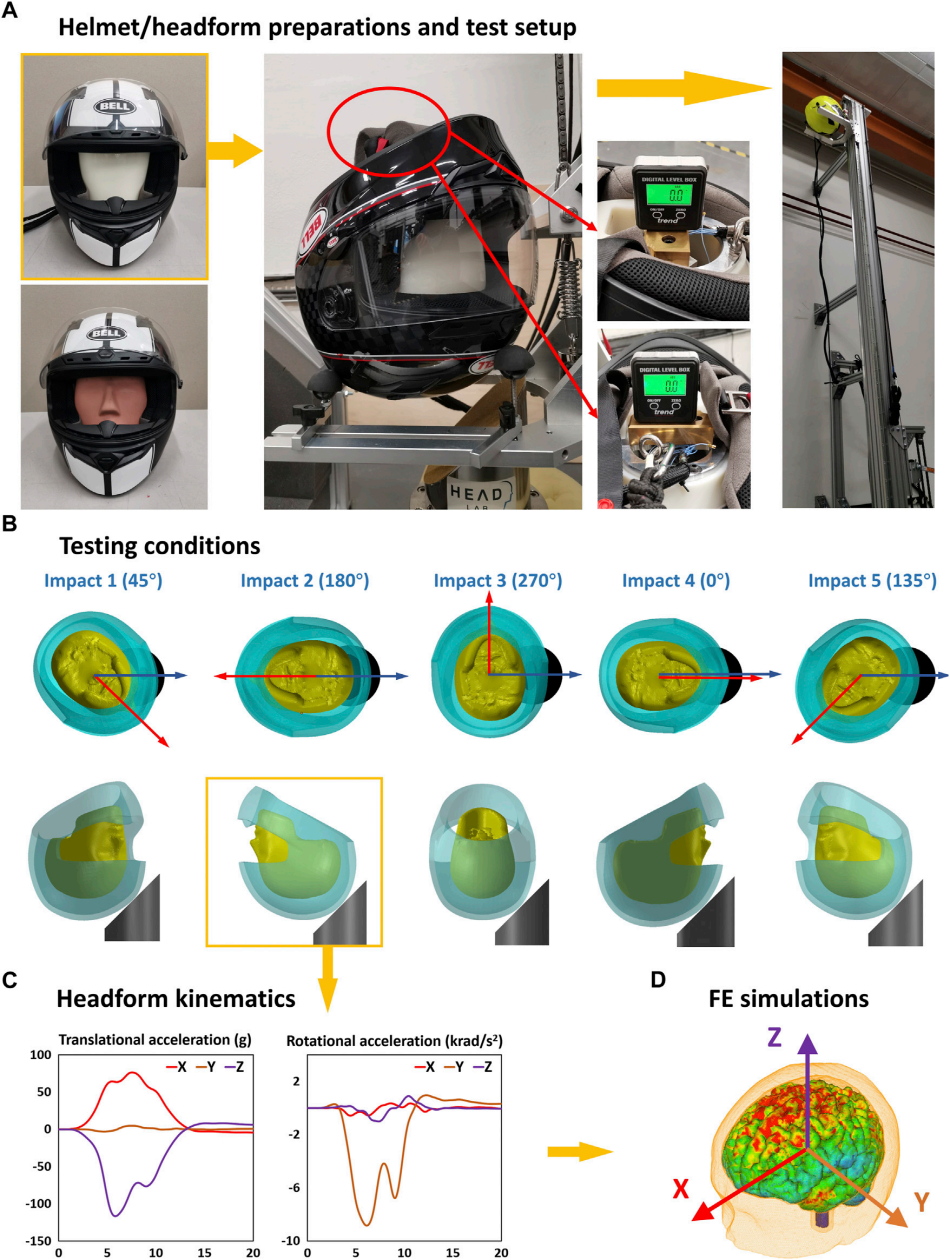
Testing and simulation methods. **(A)** Helmet/headform preparation. **(B)** Each helmet was tested at five impact points. **(C)** For each test, three translational and three rotational acceleration time-history data were recorded. **(D)** These acceleration data were applied to the Imperial College finite element model of the human head for predicting strain and strain rate.

We used the Bell Qualifier helmet (size M and a fitting head circumference of 57 cm–58 cm), purchased from the United Kingdom market. The helmet was fitted onto the headforms based on the helmet fitting requirements of ECE22.06 (Figure 2A). A load of 50 N was applied on the base of the headforms to adjust the helmet on the headforms. After placing the helmeted headforms on the platform, we used an inclinometer to adjust the headforms so that the bottom of the headforms was horizontal (< 0.5°). To prevent the movement of the helmeted headform during the falling, we used precut masking tape to hold the helmet on the platform.

The helmeted headforms were tested at five impact locations (Figure 2B). The angle between the headform’s sagittal plane and the anvil’s middle plane ranged from 0° to 270°. To minimize the accumulated damage from previous impact, the five impact points were divided into two separate helmet samples (sample 1: impacts 1–3; sample 2: impacts 4 and 5). Each impact was repeated three times at the same location using a new sample. Therefore, we performed 15 tests on 6 samples for each headform.

The helmeted headform was dropped onto a 45° anvil at a velocity of 8.0 m/s (+0.15/− 0.0) (Committee, 2020). The anvil was covered with a grade 80 abrasive paper, which was replaced after each test. We used a high-speed video camera to record the testing process. After each test, the high-speed video was checked, making sure that each test was performed as intended, and the helmet was not displaced on the platform during the free fall.

The translational and rotational accelerations of the headform (Figure 2C) were measured using a nine-accelerometer package (NAP). The accelerometers were mounted in a 3-2-2-2 array (Padgaonkar et al., 1975). The accelerometers were sampled using a datalogger at a 50 kHz frequency. We filtered the acceleration data, using a fourth-order Butterworth filter at a cut-off frequency of 1 kHz, as suggested by Ghajari et al. (2011a). We calculated the rotational velocities by integrating the rotational accelerations vs. time.

### 2.3 Finite element modeling

To evaluate the brain deformation from the tests, we used the Imperial College finite element (FE) model of the human head (Ghajari et al., 2017; Yu et al., 2022b) (Figure 2D). This model has a detailed definition of the head anatomy, including sulci and gyri, and its predictions of brain displacement have been validated against recent well-controlled experiments on postmortem human subject heads (Fahlstedt et al., 2021; Zimmerman et al., 2021). More details about the head FE model, material models and properties, model validation, and applications can be found in our previous studies (Ghajari et al., 2017; Yu and Ghajari, 2019; Yu et al., 2020; Abayazid et al., 2021; Fahlstedt et al., 2021; Yu et al., 2022c; Yu and Ghajari, 2022). The skull was modeled as a rigid body due to its small deformation in the helmeted impact. We applied the three translational and three rotational accelerations measured in the impact tests to the skull at the center of gravity of the head. The total simulation time was 30 ms, starting from the initial helmet–anvil contact. The simulations were conducted using the nonlinear hydro-code LS-DYNA R11 (Hallquist, 2007).

For each element of the brain tissue, we determined the maximum value of the first principal Green–Lagrange strain and strain rate during the simulation (we used the total time derivative of the Green–Lagrange strain tensor to determine the strain rate tensor). We further determined the 90th percentile strain and strain rate of the entire brain, which were used to evaluate the overall brain response to the impact.

### 2.4 Injury metrics and data analysis

We used four kinematics-based injury metrics and two tissue-based injury metrics to evaluate brain injury. The kinematics-based injury metrics were peak translational acceleration (PTA), peak rotational acceleration (PRA), peak rotational velocity (PRV), and brain injury criterion (BrIC) (Takhounts et al., 2013). PTA has been suggested to predict the risk of skull fracture and focal injuries (Gurdjian et al., 1966; Allsop et al., 1988). PRA has been used for predicting SDH (Gennarelli and Thibault, 1982; Depreitere et al., 2006). PRV and BrIC have been suggested to predict the risk of diffuse axonal injuries (Margulies and Thibault, 1992; Takhounts et al., 2013). The two tissue-based injury metrics were the 90th percentile values of the maximum brain strain and strain rate of the entire brain. The brain strain and strain rate are able to predict diffuse axonal injuries (Bain and Meaney, 2000; Donat et al., 2021; Hajiaghamemar and Margulies, 2021).

For each injury metric, we performed two-way ANOVA (significance level: 0.05) to determine the effects of the headform and impact location, as well as their interaction. Then, for each injury metric at each impact location, we evaluated the difference between the two headforms by conducting pairwise comparisons (post hoc).

## 3 Results

### 3.1 Headform kinematics in the oblique impact

**FIGURE 3:**
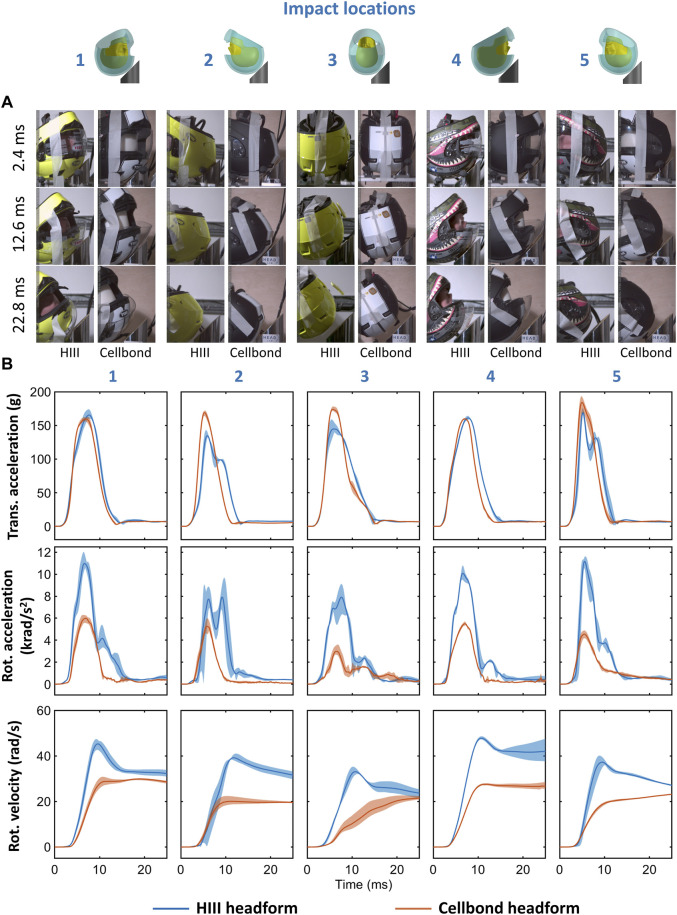
**(A)** Snapshots from the high-speed videos of the helmeted headforms under oblique impact at five different locations. **(B)** Mean resultant translational acceleration, rotational acceleration, and rotational velocity time-histories. The filled region bounds the minimum and maximum recorded traces of the three repeats for each headform at all impact locations.

Figure 3A shows the snapshots from the high-speed videos of the two helmeted headforms at five impact locations. The helmet started rolling on the anvil at around 2.4 ms and detached from the anvil at approximately 11 ms–15 ms. There was no obvious difference between helmet motion with different headforms. During the impact, the headform should have rotated inside the helmet, depending on the headform–liner friction. Due to the obstruction of the helmet, we were not able to quantify the headform motion.

Figure 3B shows the translational and rotational accelerations of the headforms in all impact locations and for three repeats. The translational acceleration of the headforms had similar peaks and shapes for impacts 1, 4, and 5. For impacts 2 (rear) and 3 (side), the translational acceleration was larger for the Cellbond headform than the HIII headform. The rotational acceleration was lower for the Cellbond headform in all impact locations. In addition, for impacts 1, 2, and 4, the duration of the rotational acceleration curve was shorter for the Cellbond headform than the HIII headform.

### 3.2 Brain strain and strain rate

**FIGURE 4:**
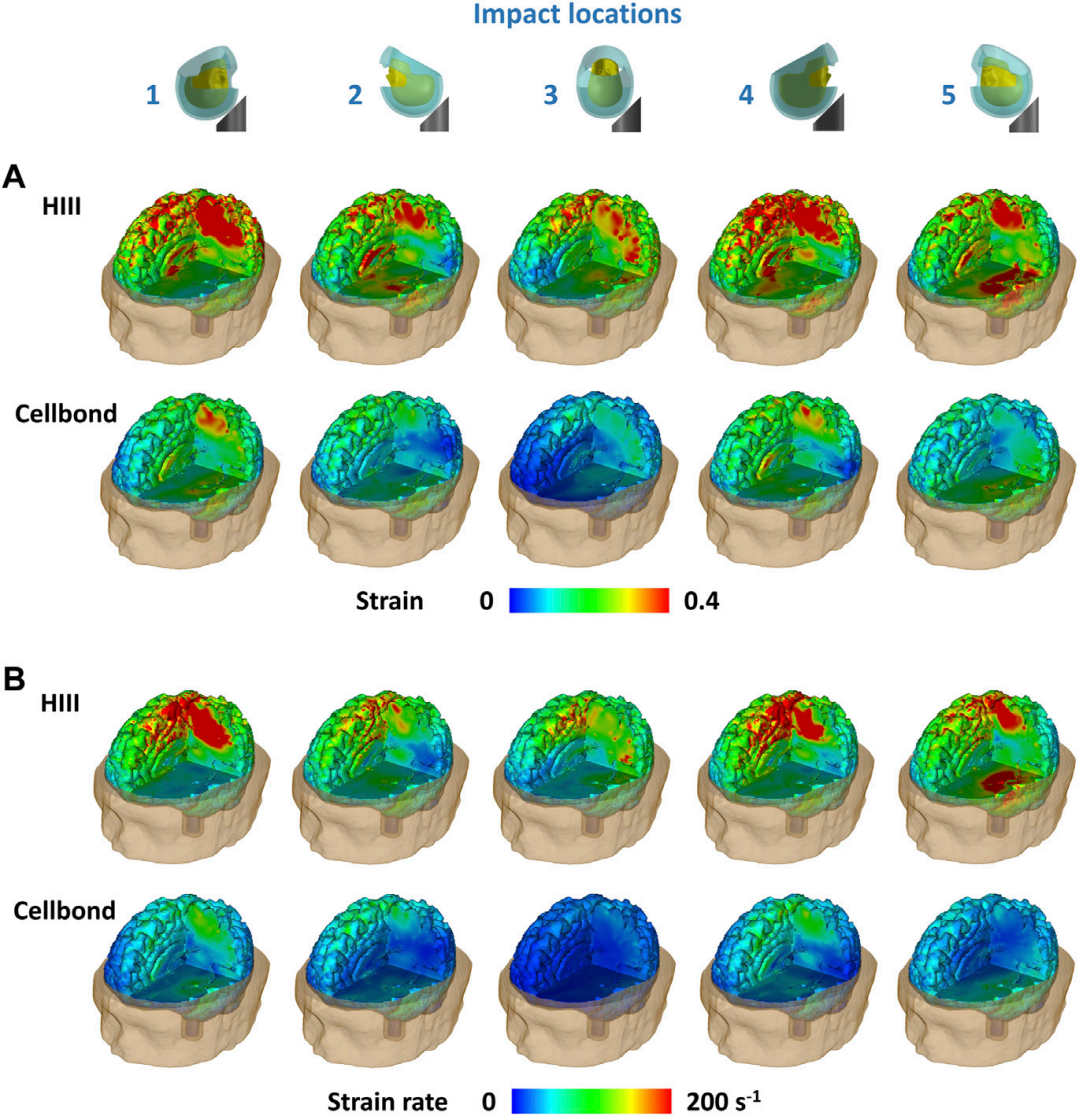
**(A)** Strain and **(B)** strain rate distribution across the brain predicted by the human head FE model.

Figure 4 shows the distribution of the first principal Green–Lagrange strain and strain rate across the brain for the two headforms under all impacts. A large volume of the brain with the HIII headform undergoes strains more than 0.4 in all impact locations when the HIII headform is used (Figure 4A). Impacts 1 and 4 (frontal impacts) produced large strains in the parietal lobe and corpus callosum. Impact 5 (rear impact) produced large strains in the parietal and temporal lobes. Impacts 2 (rear) and 3 (side) produced relatively lower strains across the brain. In contrast, when the Cellbond headform was used, only a small volume of the brain in impacts 1 and 4 underwent strains larger than 0.4.

Similar observations were made for the strain rate distribution (Figure 4B). For the HIII headform, the parietal lobe experienced a high strain rate in impacts 1 and 4, and both the parietal and temporal lobes experienced a high strain rate in impact 5. The brain underwent much lower strain rates when using the Cellbond headform.

### 3.3 The effects of the headform on brain injury metrics

**FIGURE 5:**
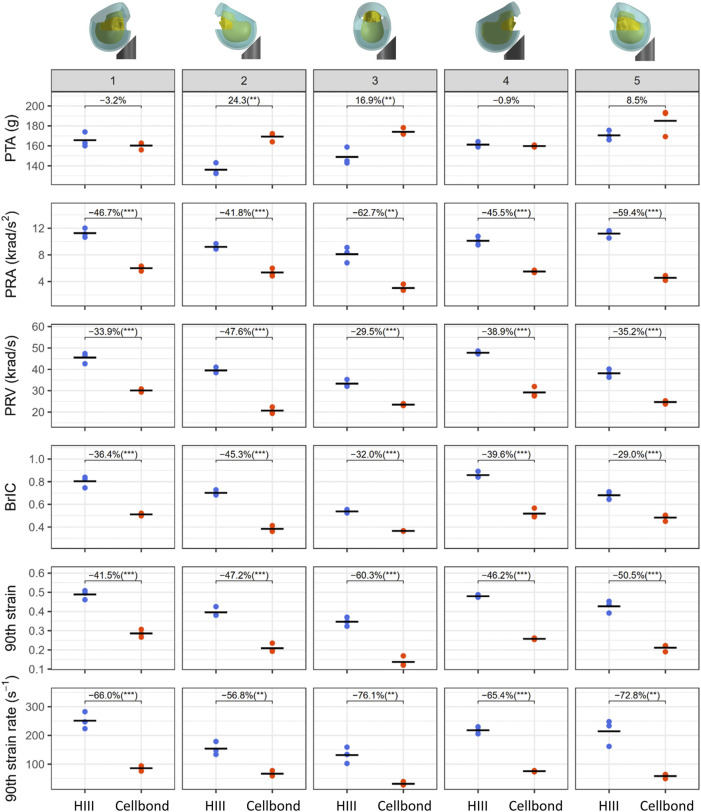
Injury metrics for the two headforms at all impact locations. The plot shows three repeats (markers) and their mean value (black line). The percentage change between the mean values of the two headforms is also shown under each impact condition (**p < 0.01 and ***p < 0.001).

Figure 5 presents all injury metrics, including the kinematics-based injury metrics (PTA, PRA, PRV, and BrIC) and the tissue-level injury metrics (the 90th percentile strain and strain rate of the brain). For each headform at each impact location, we computed the mean value and coefficient of variation (CV) of the three repeats (Table 2). Most CVs were less than 10%, showing good repeatability. Only for PRA at impact 3, both headforms produced CVs larger than 10% (HIII: 11.8%; Cellbond: 13.9%).

**TABLE 2:** Predicted injury metrics for the headforms. Mean values and CVs of the three repeats are presented.

Injury metric	Headform	Impact location				
		1	2	3	4	5
PTA (g)	HIII	166 (3.6%)	136 (3.6%)	149 (4.8%)	161 (1.4%)	171 (2.3%)
	Cellbond	160 (1.9%)	169 (2.2%)	174 (1.7%)	160 (0.5%)	185 (6.0%)
PRA (rad/s^2^)	HIII	11,268 (5.1%)	9,202 (3.7%)	8,095 (11.8%)	10,119 (5.2%)	11,198 (4.3%)
	Cellbond	6,009 (5.2%)	5,358 (8.9%)	3,021 (13.9%)	5,512 (2.7%)	4,549 (6.4%)
PRV (rad/s)	HIII	45.5 (4.6%)	39.5 (2.8%)	33.3 (4.2%)	47.8 (1.2%)	38.1 (4.1%)
	Cellbond	30.1 (2.0%)	20.7 (6.1%)	23.5 (1.6%)	29.2 (6.8%)	24.7 (2.8%)
BrIC	HIII	0.804 (5.1%)	0.702 (2.8%)	0.537 (2.3%)	0.859 (2.7%)	0.681 (4.1%)
	Cellbond	0.511 (1.9%)	0.384 (5.5%)	0.365 (0.6%)	0.519 (6.6%)	0.483 (4.9%)
90th percentile strain	HIII	0.489 (4.1%)	0.396 (5.3%)	0.347 (5.6%)	0.479 (1.3%)	0.427 (6.0%)
	Cellbond	0.286 (5.8%)	0.209 (8.9%)	0.138 (16.2%)	0.258 (1.3%)	0.211 (7.0%)
90th percentile strain rate (s^−1^)	HIII	251 (4.1%)	154 (5.3%)	132 (5.6%)	218 (1.3%)	214 (6.0%)
	Cellbond	85 (8.7%)	66 (11.9%)	31 (17.6%)	75 (2.4%)	58 (10.9%)

We conducted a two-way ANOVA, using headform and impact location as the factors. The results showed that both the headform and impact location have significant effects on all the injury metrics (*p* < 0.001). In addition, the two factors have interactions for all the injury metrics, except the brain strain.

Next, we performed a student t-test for each injury metric and impact location. The results showed that the headforms produced significantly different values under all impact conditions, except PTA. For PTA, the Cellbond headform produced significantly higher values than the HIII headform in impacts 2 and 3 only (24.3% and 16.9%, respectively).

For the rotational kinematics-based injury metrics, the Cellbond headform produced significantly lower values than the HIII headform in all impact locations. PRA with the Cellbond headform was 41.8%–62.7% lower than that with the HIII headform. This difference was slightly lower for PRV (29.5%–47.6%) and BrIC (29%–45.3%).

The Cellbond headform also led to significantly lower brain strain and strain rate than the HIII headform. For the 90th percentile strain, the Cellbond headform had 41.5%–60.3% lower values than the HIII headform. For the 90th percentile strain rate, the difference was much higher (56.8%–76.1%).

For PRA, brain strain, and strain rate, the largest difference between the two headforms was observed in impact 3 (side). For PTA, PRV, and BrIC, the largest difference was observed in impact 2 (rear). These results confirm that the headform has an interaction with the impact location in determining the value of the injury metrics.

## 4 Discussion

In this study, we used the HIII headform and Cellbond headform developed based on the CEN/TC158/WG11 work to perform the oblique impact on a motorcycle helmet. The HIII headform is often used to test helmets under oblique impact conditions, where translational and rotational kinematics of the headform is measured and used to assess the performance of helmets (Aare and Halldin, 2003; Bliven et al., 2019; Abayazid et al., 2021; Deck et al., 2021; Yu et al., 2022a). In contrast to this headform, the MoIs and CoF of the Cellbond headform are in better agreement with those obtained from measurements on live and postmortem human subjects (Trotta et al., 2018a; Connor et al., 2020). Previous research has studied helmet response with headforms that have more biofidelic MoIs (Kendall et al., 2012; Connor et al., 2020) or CoF (Trotta et al., 2018b; Juste-Lorente et al., 2021; Zouzias et al., 2021) but not a headform that has both. For the first time, this study used a headform that has both biofidelic CoF and MoIs. Our results showed that except for PTA, all other injury metrics were lower when using the more biofidelic Cellbond headform. This suggests that by using the HIII headform, we may overestimate the head rotational response during the oblique impact. These results support the development of biofidelic headforms (e.g., the Cellbond headform) for inclusion in helmet standards and rating methods to more accurately simulate the human head response in laboratory experiments.

We followed the impact conditions of the oblique impact test method in the newly issued ECE22.06 motorcycle helmet test standard (Committee, 2020). The five impact locations represent a large proportion of impact on the helmets. In addition, the impact speed (8 m/s) was also adopted from the ECE22.06 standards, which is the only test speed used in the oblique impact tests in the standard. Therefore, the testing results of our study may serve as a reference for future studies conducting ECE22.06 oblique impact tests using other headforms.

We used both kinematics-based and tissue-based injury metrics to evaluate head injury. PTA is a kinematics-based injury metric that has been suggested for predicting the risk of skull fractures and focal injuries (Gurdjian et al., 1966; Allsop et al., 1988), and it is used in all helmet standards (McIntosh and Grzebieta, 2013; Whyte et al., 2019). The Cellbond headform produced higher PTAs in rear and side impacts but similar PTAs in other impacts compared to the HIII headform. The highest PTA measured in the tests with the Cellbond headform is more than 190 g, which is higher than the 180 g limit in the linear low energy impact (6 m/s) of the ECE22.06 motorcycle helmet test standard (Committee, 2020). As PTA is correlated with the normal component of the impact velocity (Mills, 2010), increasing the normal velocity (5.66 m/s) of our oblique impact tests to 6 m/s would result in even higher PTAs. The PTA differences between the two headforms may be due to the difference in their mass and scalp materials. Such differences suggest that the risk of skull fractures of human head in real-world crashes may be underestimated when using the HIII headform.

For the metrics based on rotational kinematics of the head, i.e., PRA, PRV, and BrIC, the Cellbond headform produced significantly lower values in all impact locations than the HIII headform. PRA has been suggested as a predictor of the risk of subdural hematoma (SDH), and a 10 krad/s^2^ threshold has been suggested for it based on the PMHS experiments (Gennarelli and Thibault, 1982; Depreitere et al., 2006). When we used the HIII headform, PRA passed this threshold at three impact locations. However, when we used the Cellbond headform, PRA was less than 6 krad/s^2^ for all impact locations. PRV and BrIC are suggested to be predictors of the risk of diffuse axonal injury (DAI) (Margulies and Thibault, 1992; Takhounts et al., 2013). Margulies et al. (1990) determined a 46.5 rad/s PRV threshold for moderate to severe DAI by using physical models of human and baboon heads filled with a brain-like material and estimating shear strain deep in the brain using high-speed videography. BrIC was motivated by strong evidence for the DAI producing effects on head rotation, and it was developed by combining finite element modeling of the human head and experimental data on rhesus monkeys, baboons, and miniature pigs (Takhounts et al., 2003; Takhounts et al., 2008; Takhounts et al., 2013). BrIC has been adopted by several motorcycle helmet test standards, such as ECE22.06 and FRHP (FIM Racing Homologation Programme). A previous work has suggested a 46.5 rad/s PRV threshold for producing moderate to severe DAI (Margulies and Thibault, 1992). Using the HIII headform led to PRVs higher than this threshold in two impact locations (both frontal), but PRV was less than 32 rad/s in all impact locations with the Cellbond headform. BrIC has been included in the ECE22.06 helmet standard with a 0.78 limit, representing a 25% risk of AIS4 DAI (Takhounts et al., 2013). Again, at two frontal impacts, the HIII headform led to values larger than this threshold, while BrIC recorded with the Cellbond headform was less than 0.52 in all impacts. Due to the low shear modulus of the brain, the brain strain and strain rate are mainly produced by head rotation (King et al., 2003; Kleiven, 2013; Hajiaghamemar et al., 2020; Carlsen et al., 2021; Zimmerman et al., 2021). As a result, the brain strain and strain rate were also much lower with the Cellbond headform than the HIII headform. These results suggest that using the HIII headform overestimates the risk of brain injuries produced by head rotation.

The difference between the response of headforms under the same oblique impact conditions and using the same helmet can stem from the large difference in the coefficient of friction (CoF) at the headform/liner interface. Previous studies have shown that this CoF has significant effects on the head kinematics during oblique impact (Trotta et al., 2018b; Zouzias et al., 2021). Human skin at different regions has very different surface frictions, depending on the skin surface condition and hydration (Derler et al., 2015). Several studies have attempted to measure CoF between the skin and helmet liner or other fabric and reported different values (Zhang and Mak, 1999; Derler et al., 2015; Ebrahimi et al., 2015). The most comprehensive study on the sliding response of the human scalp under loading conditions similar to those in helmet impact was performed by Trotta et al. (2018a). Their experiments on PMHS heads showed that the scalp’s sliding response mainly includes scalp–skull sliding followed by scalp–liner sliding.

The difference between the responses of the headforms can also stem from the difference in their MoIs. A previous work has shown that MoI can have a significant effect on kinematics of the head under oblique impact conditions (Connor et al., 2018). The emphasis of the HIII design has been on fidelity in the frontal impact, which may explain why its lateral MoI is close to that of the human head but not the other MoIs (Yoganandan et al., 2009). In the design of the Cellbond headform, this aspect has also been improved. We showed that the MoIs of this headform are close to those obtained by analyzing the CT images of a large cohort of living adults (relative difference: I_xx_: 10.5%, I_yy_: 2.2%, and I_zz_: 2.6%). The relative differences are similar or smaller than those between the 3D-printed headform and human head (I_xx_: 10.2%, I_yy_: 2.5%, and I_zz_: 16.1%) (Connor et al., 2018). Therefore, the Cellbond headform can better represent the MoIs of an average (western) male rider than the HIII, EN960, and 3D-printed headforms in previous studies (Connor et al., 2018; Juste-Lorente et al., 2021).

We found that the impact location influences the relationship between headform and injury metric. For example, the HIII headform produced the lowest PTA at impact 2, while for the Cellbond headform, impacts 1 and 4 produced the lowest PTA. For PRV, the lowest values occurred at impact 3 for the HIII headform and at impact 2 for the Cellbond headform. These differences may be explained by the different MoIs about the rotation axes dominant at different impact locations. Most previous studies included impact 3 (lateral) and impact 4 (frontal) but not impacts 1, 2, or 5, in their oblique impact tests (Trotta et al., 2018b; Connor et al., 2018; Abayazid et al., 2021; Juste-Lorente et al., 2021). Interestingly, our results showed that impact 5 produced the highest PTA, and impact 1 produced the highest PRA for both headforms. Hence, our results support the inclusion of different impact locations and injury metrics in future helmet test methods that measure both translational and rotational kinematics of the head.

The gold standard for testing the fidelity of headforms is using human head kinematics under different impact conditions, but such data are currently unavailable. Alternatively, postmortem human subjects’ (PMHS) head impact data can be used (Hodgson, 1975; Mertz, 1985; Loyd et al., 2014; Bonin et al., 2017). A previous study conducted normal impact tests with bare adult PMHS heads and HIII headform (Loyd et al., 2014) and did not find a significant difference between PTA measured with the PMHS heads and HIII headform. It should, however, be noted that skull deflection and skin compression can play significant roles in determining PTA in bare head impact but not in helmeted head impact. A more recent study performed normal impact tests on helmeted PMHS heads and helmeted HIII headform (Bonin et al., 2017). They found that the PMHS head produces higher PTA than the HIII headform, consistent with our findings when comparing the Cellbond and HIII headforms. Although data from impact on the helmeted PMHS head can be useful in further testing the fidelity of headforms, they have some limitations. One limitation is an accurate recording of both translation and rotational accelerations given that the helmet would cover a large part of the head, thus leaving few places for installing sensors. Another limitation is the cost of PMHS tests, which limits the number of experiments. The MoIs used to design the new headform are based on CT scans of 56 adults, a number that is hard to achieve with PMHS tests and not available in the current literature. Future works may focus on producing kinematics data from normal impact and oblique impact on helmeted PMHS heads, which can be used to further test the fidelity of new headforms. It should, however, be emphasized that the Cellbond headform has more realistic CoF and MoIs than other headforms and as such is expected to provide more realistic kinematics responses.

This study has some limitations. We compared the performance of the Cellbond headform with that of the HIII headform only. We chose the HIII headform because it is widely used in helmet oblique impact tests and helmet rating methods (Stigson et al., 2017; Bliven et al., 2019; Deck et al., 2021; Yu et al., 2022a). A previous study showed that the EN960 headform produced lower rotational acceleration and velocity than the HIII headform under oblique impact conditions (Connor et al., 2018). However, the differences between the two headforms depend on test speed and helmet type (equestrian helmet), which are both very different from our study. Therefore, we were not able to use the findings to estimate the performance of the EN960 headform in our study. Future works should use the current test condition and a motorcycle helmet to test EN960 and other commonly used headforms, such as NOCSAE. We used an isolated headform without the neck, similar to previous studies on helmet impact (Halldin et al., 2001; Stigson et al., 2017; Trotta et al., 2018b; Connor et al., 2018; Zouzias et al., 2021). It has been shown that the neck has an effect on the head response, and this effect varies depending on the loading direction (Nightingale et al., 1996; Ghajari et al., 2011a; Ghajari et al., 2011b, 2013; Abayazid et al., 2021). However, considering that the impact duration in our study is short (approximately 15 ms) and the available dummy necks have limitations when loaded under head-first impact conditions, we did not include a neck in the test. The Cellbond headform’s external shape was based on the EN960-J headform. This decision was supported by a previous study that measured the head dimensions of more than 500 young adults and motorcyclists (Gilchrist et al., 1988). It was shown that the dimensions (e.g., the circumference and breadth, etc.) of the EN960-J headform were close to those determined from the regression of human data. However, these basic dimensions may not be adequate to define a biofidelic 3D headform shape. In addition, some anatomical features, such as the ear and nose, have been simplified. When previous studies have suggested that the headform shape can affect the headform kinematics (Bonin et al., 2017; Connor et al., 2018), future works should investigate this effect and further improve the biofidelity of the Cellbond headform’s shape.

Another limitation is that we tested only one helmet type. More helmet types (e.g., bicycle helmets) should be included in the future tests as helmet geometry and materials can have a large influence on the helmet performance under impact conditions. Moreover, we tested the helmets using one impact velocity with equal tangential and normal components with respect to the anvil. A recent study has shown that the tangential speed significantly affects the rotational head motion but not linear motion (Juste-Lorente et al., 2021). Future works should extend this study by varying the tangential speed. In addition, we used the five impact locations defined in the ECE22.06 standards. These impact locations are obtained by rotating the headform within the transverse plane, and hence they lead to small head accelerations about the Z-axis. More impact locations should be included in future tests, particularly those that produce larger rotations about the Z-axis. Finally, the Cellbond headform used in this study has a hard surface and cannot model the elasticity of the human scalp, and hence it may overestimate PTA (Trotta et al., 2018b). Future works should focus on the development of headforms with a more biofidelic scalp.

In summary, we studied the response of the HIII headform and Cellbond headform under helmeted oblique impact conditions. The latter headform has more biofidelic MoIs and CoF, and it is an early version of the headforms under development by working group 11 of CEN/TC158. We showed that the more biofidelic Cellbond headform produced similar or higher translational acceleration than the HIII headform but significantly lower injury metrics based on head rotation and predicted strain and strain rate across the brain. This indicates that the HIII headform may overestimate the risk of brain injuries produced by head rotation. It may also not adequately assess and distinguish the performance of helmet technologies developed for mitigating such injuries. We, therefore, recommend adopting headforms with biofidelic MoIs and CoF (e.g., the Cellbond headform) in future helmet standards and rating methods to better assess the protection performance of helmets.

## Data Availability

The raw data supporting the conclusion of this article will be made available by the authors, without undue reservation.
